# Exercise Does Not Protect against MPTP-Induced Neurotoxicity in BDNF Happloinsufficent Mice

**DOI:** 10.1371/journal.pone.0043250

**Published:** 2012-08-17

**Authors:** Kim M. Gerecke, Yun Jiao, Viswajeeth Pagala, Richard J. Smeyne

**Affiliations:** 1 Department of Psychology and Neuroscience Program, Rhodes College, Memphis, Tennessee, United States of America; 2 Department of Developmental Neurobiology, St. Jude Children’s Research Hospital, Memphis, Tennessee, United States of America; 3 Hartwell Center for Bioinformatics, St. Jude Children’s Research Hospital, Memphis, Tennessee, United States of America; Virginia Commonwealth University, United States of America

## Abstract

Exercise has been demonstrated to potently protect substantia nigra pars compacta (SN) dopaminergic neurons from 1-methyl-4-phenyl-1,2,3,6-tetrahydropyridine (MPTP)-induced neurotoxicity. One mechanism proposed to account for this neuroprotection is the upregulation of neurotrophic factors. Several neurotrophic factors, including Brain Derived Neurotrophic Factor (BDNF), have been shown to upregulate in response to exercise. In order to determine if exercise-induced neuroprotection is dependent upon BDNF, we compared the neuroprotective effects of voluntary exercise in mice heterozygous for the BDNF gene (BDNF+/−) with strain-matched wild-type (WT) mice. Stereological estimates of SNpc DA neurons from WT mice allowed 90 days exercise via unrestricted running demonstrated complete protection against the MPTP-induced neurotoxicity. However, BDNF+/− mice allowed 90 days of unrestricted exercise were not protected from MPTP-induced SNpc DA neuron loss. Proteomic analysis comparing SN and striatum from 90 day exercised WT and BDNF+/− mice showed differential expression of proteins related to energy regulation, intracellular signaling and trafficking. These results suggest that a full genetic complement of BDNF is critical for the exercise-induced neuroprotection of SNpc DA neurons.

## Introduction

Exercise confers substantial health benefits, including potent protection of the brain against age-related functional decline and a wide range of neurological insults. For example, aerobic exercise in humans prevents normal age-related declines in the volume of cortical grey matter [1] and cognitive abilities [2]–[5]. Exercise has also been shown to decrease the risk for developing Alzheimer’s disease [6], [7] and Parkinson’s disease (PD) [8]. In addition, exercise has also been reported to slow the progression of these disorders [9], [10].

While neuroprotective and neurorestorative effects of exercise have been demonstrated in humans, modeling these disorders in animals is necessary to discern the mechanism(s) that underlie this effect. Both voluntary and forced exercise [11]–[14] protects against the SNpc dopaminergic (DA) neuron loss that results from acute or chronic exposure in the 1-methyl-4 phenyl-1, 2, 3, 6-tetrahydropyridine (MPTP) model of Parkinson’s disease [15], [16], as well as in other models of PD [13], [14], [17]–[19]. Thus, exercise has been shown to potently protect against neurotoxin-induced DA neuron death in the SN.

The process by which MPTP acts in the brain offers clues to its pathological mechanisms. Upon entry into the CNS, MPTP is metabolized to the reactive molecule 1-methyl-4-phenyl 1–2, 3-dihydropyridium (MPP^+^) [20] which acts to interfere with Complex I respiration in the electron transport chain of the mitochondria [21]–[23], starving DA neurons of required energy, and ultimately, killing them [24]. In addition, MPTP induces the activation and proliferation of microglia [25]–[27] which subsequently generate reactive oxygen species [28]–[30]. Taken together, these studies suggest that in addition to directly killing neurons by interfering with energy metabolism, the concomitant microglial activation and induction of oxidative stress is fundamental to the progression of MPTP-induced DA neurodegeneration.

Several molecular mechanisms have been hypothesized to explain how exercise protects DA neurons from oxidative and inflammatory stress, most notably the upregulation of trophic factors. Exercise increases the expression of transcripts for brain-derived neurotrophic factor (BDNF) in the hippocampus [31]–[34] that is sustained over time [35], and the magnitude of this change correlates directly with the amount of physical activity [31]. Exercise has been shown to protect SNpc DA neurons from death induced by exposure to the bacterial endotoxin lippopolysaccharide (LPS) via increases in BDNF [19]. In addition to its role in neuroprotection, BDNF has been shown to promote the growth and survival of DA neurons [36] and decrease the production of free radicals [37]–[40]. Based upon these varied BDNF-mediated effects, exercise-induced increases in this trophic factor may act directly on neurons to strengthen them against toxic insult, as well as indirectly through inhibition of oxidative stress.

To determine if BDNF is critical for exercise induced neuroprotection, we examined the effects of exercise in mice carrying a heterozygous deletion of the BDNF gene [41]. These transgenic mice are viable, and despite the fact that they only express between 40–60% expression of the WT levels of BDNF in the CNS [42], [43], show relatively normal brain development [42], [44], [45]. In the basal ganglia, BDNF+/− mice have normal levels of serotonin, norepinephrine and dopamine [42] and do not differ from WT mice in their expression of a number of dopaminergic cell markers including tyrosine hydroxylase (TH), dopamine transporter (DAT), and the vesicular mediated transporter 2 (VMAT2) [44], [46]. Importantly, BDNF +/− mice do not differ from their WT littermates in measures of spontaneous or general locomotor activity [47]–[49].

Here, we demonstrate that exercised BDNF+/− mice are not protected from MPTP-induced neurodegeneration of SNpc dopaminergic neurons. A proteomic analysis of the STR and SN of WT and exercised BDNF+/− mice suggests changes in proteins involved in cellular metabolism, bioenergetics, and intracellular signaling may underlie the lack of exercise mediated neuroprotection in BDNF+/− mice.

## Materials and Methods

### Animals

This study was carried out in strict compliance with the recommendations in the Guide for the Care and Use of Laboratory Animals of the National Institutes of Health. The protocol was approved by St. Jude Children’s Hospital’s Institutional Animal Care and Use Committee (Protocol Number: 364). These experiments were carried out in accordance with The Code of Ethics of the World Medical Association (Declaration of Helsinki) for animal experiments. Female mice containing a heterozygous deletion of the BDNF gene [41] on a C57BL/6J background (B6.129S4-Bdnf<tm1Jae>/J; Jackson Laboratory, Bar Harbor, Maine) were purchased and bred in our vivarium with C57BL/6J (WT) males (Jackson Laboratory). All mice were maintained in a temperature-controlled environment with free access to food and water and kept on a 12-h light/dark cycle from 7AM to 7PM each day.

Housing conditions were as described previously [16]. Briefly, 4–6 week old WT or BDNF+/− mice in the Exercise (Ex) condition were housed in computerized “Wheel Cages” from Lafayette Instruments (Lafayette, IN, Model 80820) that monitored individual running activity. Each mouse in Ex housing had unrestricted access to an exercise wheel throughout the entire experimental period. For standard housing (SH), mice were housed individually in standard 12×8 inch polycarbonate mouse cages.

### Genotyping

At time of weaning (3–4 weeks) the tails of the mice were anesthetized on ice and the tips removed. Total DNA was isolated from the tail tissues as follows: Tail samples were incubated overnight at 55°C in sterile extraction buffer (50 mM Tris Base, 25 mM EDTA, 100 mM Nacl, 1% v/v Triton-X 100, 0.5 mg/ml Proteinase K). Following incubation, the samples were vortexed, and to the tubes 500 µl of Tris-equilibrated phenol (pH 8.0)/cholorform/isoamyl alcohol (25∶24:1) was added to each tube. Samples were then vortexed for 20–30 seconds and spun for 5 minutes at maximum speed in a microcentrifuge at room temperature (RT). The aqueous phase was then removed to a new tube, and to this 500 µl of Chloroform/isoamyl alcohol (24∶1) was added to each tube. Following another vortex for 20–30 seconds, tubes were again spun for 5 minutes at maximum speed in a microcentrifuge at RT. Again the aqueous phase was removed to a new tube, and to this 50 uL 3 M sodium acetate and 1 mL 100% ethanol (EtOH; at −20°C) was added to each sample following which they were incubated for 10 minutes on ice, or stored overnight at −20°C. Samples were next spun for 15 minutes at maximum speed at 4°C to pellet the DNA. The supernatant was removed and discarded, and the pellets were washed with 70% EtOH. The pellets were then spun for 5 minutes at maximum speed and at 4°C and the EtOH removed. Pellets were then dried under vacuum for 3–5 minutes and solubilized in 200 µL sterile dH2O by incubation either at 37°C for 15 minutes or at 4°C overnight. DNA extracts were stored at −20°C.

**Figure 1:**
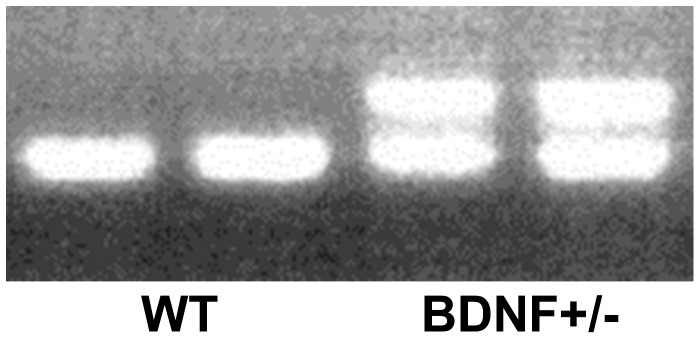
Representative gel of PCR products for genotyping. WT animals were identified by the presence of one band at ∼275 base pairs, while BDNF+/− mice were identified by the presence of both the ∼275 base pair band and a unique band at ∼340 base pairs.

Genotype was determined by PCR of tail DNA using a protocol modified from that of Jackson Laboratories (www.jaxmice.jax.org). Two microliters of each DNA sample was added to 23 µl of master mix (1X PE buffer II/2mM MgCl_2_/0.2 mM dNTP/0.69 mM DNA dye/5 U/µl Taq Polymerase [Promega, Madison, WI]/48% sterile dH_2_0) containing 2 µm of each primer (Hartwell center for Bioinformatics, St. Jude’s Children’s Research Hospital, Memphis, TN). Primers sequences were (5′- 3′): Common (ATG AAA GAA GTA AAC GTC CAC); wt reverse (CCA GCA GAA AGA GTA GAG GAG); reverse (GGG AAC TTC CTG ACT AGG GG). PCR was performed using the following cycles: 94°C for 3 minutes, 32 cycles of [94°C for 30 seconds 55°C for 1 minute], 72°C for 1 minute], 72°C for 2 minutes 4°C ∞. PCR products were then mixed with loading dye (30% glycerol/0.5% cresol red/70% dH_2_O) resolved on a 1.5% agarose gel containing 0.01% ethidium bromide by electrophoresis (100 v, ∼20 minutes). BDNF+/− mice were identified by the presence of two bands, one at ∼275 bp that was common with Wt mice, and another one at ∼340 bp that is unique for the heterozygous mice (Figure 1).

### MPTP Administration

The day prior to administration of MPTP, all mice were placed individually in SH cages on warming pads (set to “Off”) in a biosafety cabinet. Mice were administered either vehicle [0.9% sterile saline (Sal)] or MPTP (Sigma, St.Louis, MO) using the acute protocol 4×20 mg/kg ip injections at 2-h intervals) [50]. Beginning on the morning of the day of the injections, the warming pads were turned on to abrogate the 2–3°C reduction in core temperature that occurs with MPTP administration [51]; in our laboratory we have found that the use of the warming pads for 72 hours improves survival of the animals. To ensure continuity between experiments by minimizing possible seasonal changes in running patterns, these experiments were repeated 3 times over the course of a year and the data was compiled in the final analysis.

### Immunohistochemistry

Seven days following MPTP administration, a time that at which SNpc DA neuron loss is complete [52], [53], mice were deeply anesthetized with tribromoethanol (250 mg/kg (i.p) and transcardially perfused with 0.1 M phosphate buffered saline (PBS; pH 7.4) followed by 4% paraformaldehyde. Brains were removed from the calvaria and processed for immunohistochemistry as previously described [16]. Briefly, brains were sectioned at 10 microns and mounted on polyionic slides (Superfrost plus, Fisher Scientific). Standard immunhistochemical techniques using a polyclonal antibody directed against tyrosine hydroxylase (TH) (1∶250 in blocking buffer; Pel Freez, Rogers, AR) were to identify dopaminergic neurons in the SNpc as previously described [54].

### DA Cell Quantification and Analysis

Estimates of the number of dopaminergic neurons in the SNpc were made using previously validated stereological methods [55] used in many PD models (including MPTP, rotenone, paraquat and 6-hydroxydopamine models) in rodents [16], [27], [56]–[70] non-human primates [71]–[77], as well as in humans [75]. Statistical analyses were performed using ANOVA followed by LSD post-hoc comparisons (SPSS v16.0 software), and statistical significance was set to *p*<0.01. The numbers of mice in this analysis were: WT SH+Sal n = 8, WT SH+MPTP n = 10, BDNF+/− Ex Sal n = 4, BDNF+/− Ex+MPTP n = 8, WT Ex+MPTP n = 6.

### Running Activity Analysis

Initially, daily running behavior was averaged within genotypes and plotted over the 90 day duration of the experiment. Because there appeared to be differences in the initial running behavior between WT and BDNF+/− mice, a repeated measures ANOVA with a Greenhouse-Geisser correction was conducted to compare the effect of genotype on running distances over the duration of the experiment. For an initial comparison, daily running totals were analyzed in 30 day periods (d1–30, d31–60, d61–90). For all analyses, the within-subjects factor was the day of the experiment, the between-subjects factor was genotype (WT n = 10; BDNF+/− n = 11), and a p<0.05 was considered significant.

### 2-D Gel Electrophoresis and Analysis

For proteomic analyses (n = 3 for each group), WT and BDNF+/− mice were allowed to exercise 3 months, after which time the animals were anesthetized with tribromoethanol (250 mg/kg, i.p.), the brains quickly removed, and the striatum (STR) and substantia nigra (SN) were isolated by dissection. All tissue processing, image acquisition, analysis, and protein identification by mass spectrometry were performed as previously described [16]. Results of pair-wise image comparisons in which one gel is designated as the reference and the other as the test sample are reported in terms of fold-increase/decrease in the normalized volume of matched spots. For all comparisons p<0.05 and a fold change of ±1.5 was considered significant. In addition, post-translationally modified proteins, indicated by spots that shifted positions or twin spots present in only one condition, were also included for identification. Protein assignments are made on the basis of both MS and MS/MS spectra. At all stages of post-electrophoretic sample processing, rigorous procedures were employed to minimize sample contamination.

## Results

### Analysis of Running Activity in BDNF+/− and WT Mice

**Figure 2:**
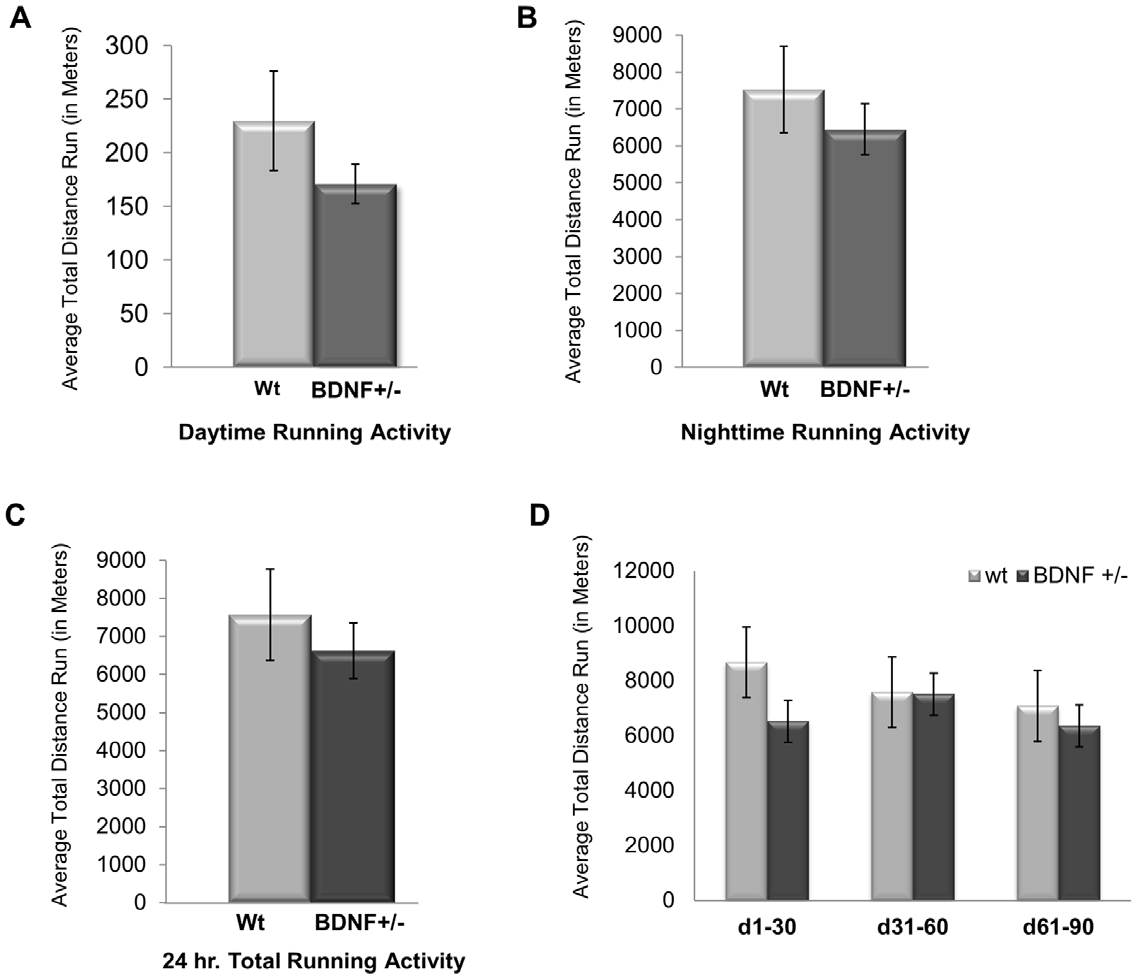
WT and BDNF+/− mice do not differ in the amount of running. Average total running activity for WT and BDNF+/− mice during the (A) daytime, (B) nighttime or (C) the total 24 hour interval. (D) The average total running distance for WT and BDNF+/− mice at 30 day intervals. Bars indicate the average of total running activity ± SEM.

We have previously shown that C57BL/6J mice run, on average, approximately 7.5 km per 24 hour period [16]. Since BDNF+/− mice have been reported to show deficits in specific task-related motor behaviors [42], [47], [78], we empirically determined if BDNF+/− mice ran less than their WT littermates. When comparing the average total distance run, no differences were observed between WT and BDNF+/− mice in either their daytime (Figure 2A), nighttime (Figure 2B), or 24 hour (Figure 2C) activity levels. WT mice ran an average of 19,148.37±2995.59 revolutions/24 hour period while BDNF +/− mice averaging 16,728.83±1826.19 revolutions/24 hour period (Figure 2C). Both the WT and BDNF+/− mice were most active during the nighttime hours (WT  = 95.19%; BDNF+/−  = 97.42%). Thus, there was no discernable difference between the average total amount and day/night pattern of running between WT and BDNF+/− mice.

**Figure 3:**
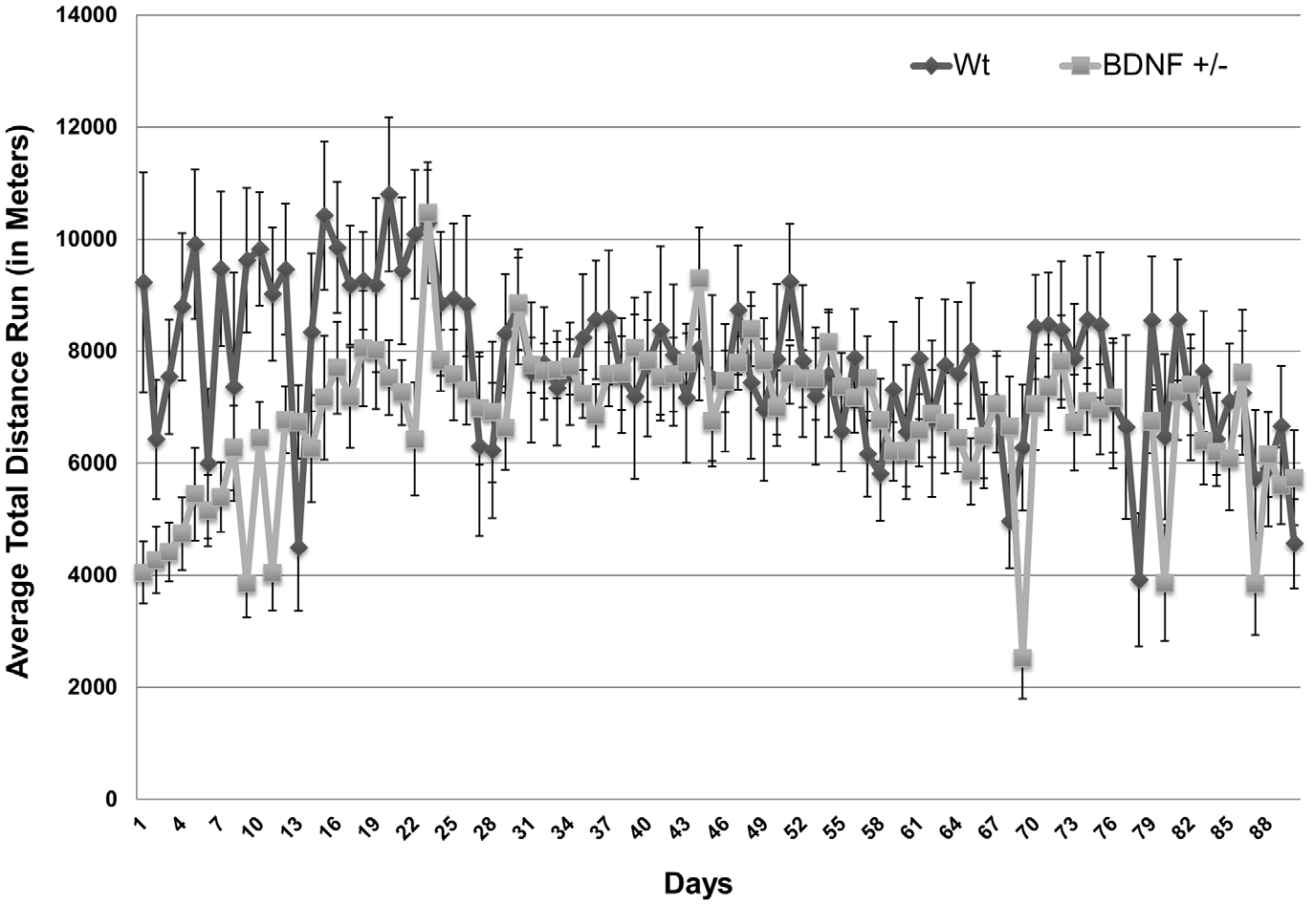
WT and BDNF+/− mice do not differ in the pattern of running. The pattern of average running activity for mice over the duration of the 90-day experimental period. Points represent the average total running activity for WT or BDNF+/− mice for each 24 hour period ± SEM.

The overall activity patterns of WT and BDNF+/− mice were also similar over the course of the entire experimental period. Both WT and BDNF+/− mice show high levels of activity (measured as distance run) that plateaus at around the 4^th^ week (Figure 3). From week 4 through the duration of the 90 day experiment the distance run decreases to a level 85% of that for the first four weeks, and then remains consistent (Figures 2D, 3).

### DA Neuron Analysis

**Figure 4:**
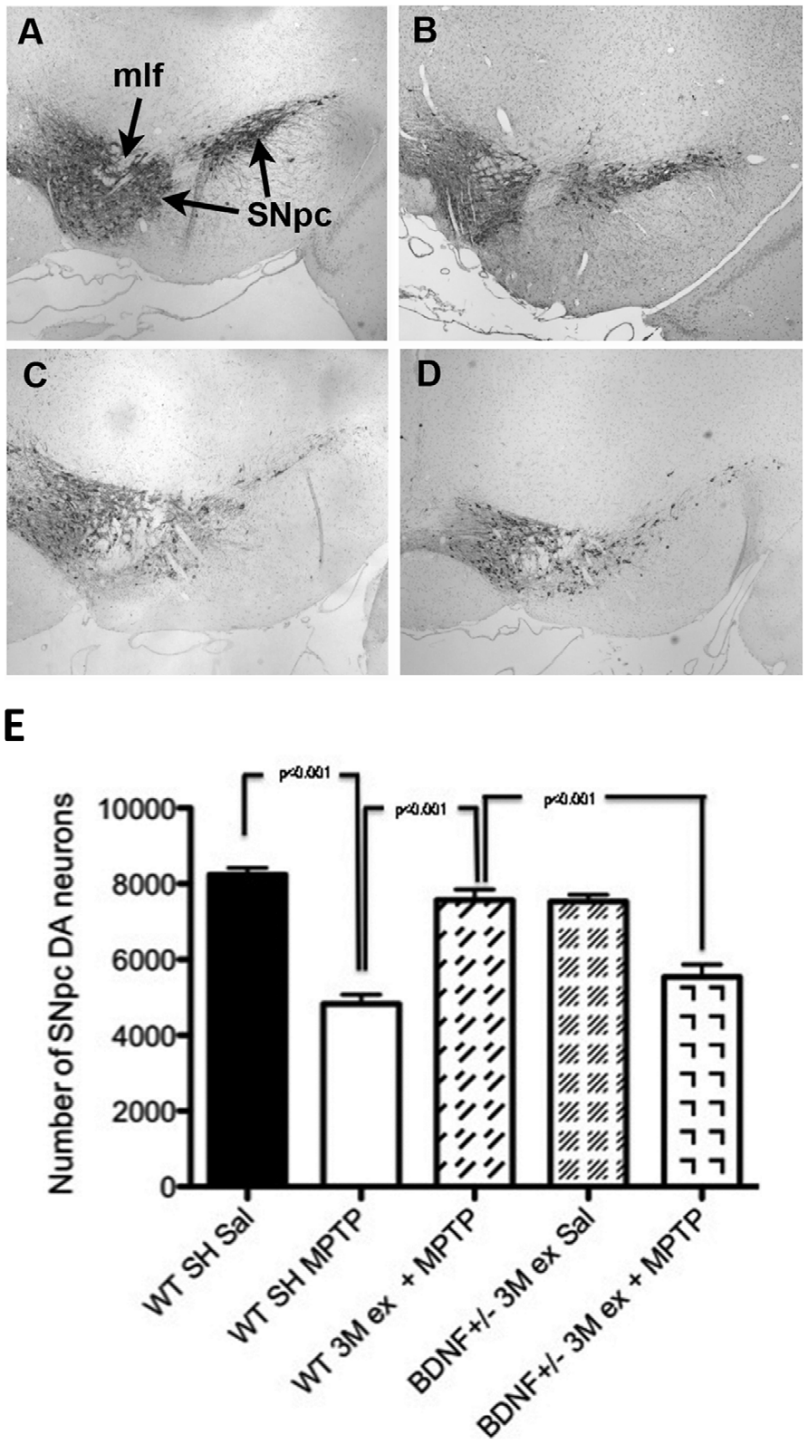
Exercise does not protect against MPTP-induced SN DA neurotoxicity in BDNF +/− mice. (A) Representative photomicrograph of the SNpc of C57BL/6 mouse at the level of the medial longitundinal fasciculus (mlf). (B) Representative photomicrograph of the SNpc of BDNF+/− mouse at the level of the medial longitundinal fasciculus (mlf). (C) Representative photomicrograph of the SNpc of the C57BL/6 mouse 7 days following MPTP mouse at the level of the mlf. (D) Representative photomicrograph of the SNpc of the BDNF+/− mouse allowed 3 months exercise and then treated with MPTP. There is no difference in the appearance of the sedentary MPTP-treated C57BL/6 mouse and the BDNF+/− mouse allowed exercise. (E) BDNF+/− mice allowed free access to running wheels for 90 days prior to MPTP administration lost significantly greater SN DA neurons than exercising WT littermates. Bars represent the average ± SEM; * p<0.01 as compared to WT SH+Sal; + p<0.01 as compared to WT Ex+MPTP.

We have previously demonstrated that 3 months of unrestricted voluntary wheel running significantly protected SN DA neurons from MPTP-induced death [16]. To determine if BDNF+/− mice were similarly protected, WT and BDNF+/− littermates were allowed unrestricted access to running wheels for 3 months (Ex) prior to administration with either saline (Sal) or MPTP. Examination of baseline SNpc DA neuron number in BDNF+/− mice showed no significant differences from their WT littermates (Figure 4E). As shown previously, exercise completely protected against MPTP-induced DA cell loss in the SN of WT mice as there was no statistically significant difference in DA neuron counts between WT sedentary mice administered saline (Figure 4A) compared to WT exercised mice that received MPTP (Figure 4B, E). However, for mice deficient in BDNF exercise did not confer DA neuroprotection (Figure 4D). In these mice, MPTP-induced a statistically significant 26.49% DA cell loss in the SN compared to exercised BDNF+/− mice administered saline (Figure 4E). The magnitude of MPTP-induced SN DA neuron loss in exercised BDNF+/− mice (Figure 4C) was equal to that seen in sedentary WT mice administered MPTP (Figure 4D, E). These results indicate that 90 days of voluntary wheel running exercise does not protect against MPTP-induced DA neurotoxicity in the SN of mice deficient in BDNF.

### Proteomic Analysis

We have shown previously that exercise induced changes in the expression of proteins involved in energy metabolism, glycolysis, and amino acid transport/metabolism [16]. Since BDNF+/− mice did not show any exercise-induced neuroprotection, we used 2D gel electrophoresis to identify differential expression of proteins in the SN and STR between WT and BDNF+/− mice allowed 90 days of unrestricted running. We found changes in the expression levels of 21 proteins while two proteins showed a modification of phosphorylation. Most of the protein changes were detected in the striatum (Table 1).

**Table 1:** Protein changes identified by 2D gel electrophoresis following 3 months of exercise in the substantia nigra and striatum of BDNF +/− and Wt mice.

Description	Accession #		Genotype		BrainRegion		*p*-value		Change
***Cytoskeleton***									
internexin neuronal intermediate filament protein, alpha (Ina)	17390900		BDNF+/−		SN		0.016		Dec 1.7
***Vesicle Trafficking and Neurotransmitter Release***									
Guanosine diphosphate dissociation inhibitor 2 (GDI2)	33244009		BDNF+/−		STR		0.031		Inc 1.70
Neurocalcin delta (NCALD)	15029877		BDNF+/−		STR		0.045		Inc 1.50
N-ethylmaleimide sensitive fusion protein attachment protein alpha	133385392		BDNF+/−		STR				Modified
***Energy Metabolism***									
NADH dehydrogenase (ubiquinone)	23346461		Wt		SN		0.003		Inc 2.35
ATP synthase, H+ transporting, mitochondrial	21313679		BDNF+/−		SN		0.006		Dec 1.49
			BDNF+/−		STR		0.015		Dec 2.00
***Glycolysis***									
Dihydrolipoamide S-acetyltransferase (E2 complex)	47125065		BDNF+/−		SN		0.029		Dec 1.60
M2-type pyruvate kinase	1405933		BDNF+/−		STR		0.008		Dec 1.49
Enolase 1/Alpha enolase	54673814		BDNF+/−		STR		0.003		Dec 1.80
Phosphoglycerate mutase1 (PGM1)	12963669		BDNF+/−		STR		0.005		Inc 1.90
***Amino Acid Transport, Synthesis and Metabolism***									
Biliverdin reductase B (flavin reductase NADPH)	21450325		BDNF+/−		STR		0.009		Inc 1.50
Proteasome (prosome, macropain) subunit, beta type, 7)(Psmb7)	29351589		BDNF+/−		STR		0.000		Inc 1.68
Proteasome (prosome macropain), alpha type 4 subunit	6755196		BDNF+/−		STR		0.016		Inc 1.48
Pyrimidine nucleoside monophosphate kinase (UMP-CMP kinase)	33150592		BDNF+/−		STR		0.003		Inc 1.45
Glutathione S-Transferase Yfyf Cys, 47-Carboxymethylated Class Pi, Chain B	2624496		BDNF+/−		STR		0.007		Inc 1.90
***Cytoplasmic Signaling Molecules and Regulatory Factors***									
Phosphotyrosyl phosphatase activator (PTPA)	4486428	Wt		SN		0.030		Inc 2.03	
Protein Phosphatase 3/Calcineurin	13277370	Wt		SN				Modified	
Map kinase kinase 1 (MAPKK1)	13928886	BDNF+/−		STR		0.006		Inc 1.57	
Dual specificity phsophatase 3 (DUSP3)	21312314	BDNF+/−		STR		0.013		Inc 1.50	
14-3-3 protein epsilon	67464424	BDNF+/−		STR		0.038		Inc 4.20	
Peptidylprolyl isomerase A (PPIA)/cyclophillin A	48145531	BDNF+/−		STR		0.037		Inc 1.70	
DnaJB6	53734660	BDNF+/−		STR		0.013		Inc 2.20	
Cytosolic acyl coenzyme A thioester hydrolase	28376965	BDNF+/−		STR		0.029		Dec 1.80	

Five proteins known to function in the glycolysis and TCA cellular energetic pathways were decreased in the SN and STR of BDNF+/− mice compared to WT mice. These include the M2-type pyruvate kinase, enolase 1/Alpha enolase, the respiratory pathway enzyme ATP synthase, and dihydrolipoamide S-acetyltransferase. Phosphoglycerate mutase 1 (PGM1), increased in the STR of BDNF+/− mice. NADH dehydrogenase/ubiquinone, the first enzyme in mitochondrial electron transport chain [79], was increased in the SN of WT mice allowed unrestricted access to running wheels.

We also observed changes in the relative abundance of five proteins that function in amino acid transport, synthesis and metabolism in BDNF+/− as compared to WT mice. In the STR of exercised BDNF+/− mice we found an increase in the expression of flavin reductase NADPH/biliverdin reductase B, the alpha type 4 and beta type 7 subunits of the proteasome (prosome, macropain), and pyrimidine nucleoside monophosphate kinase [UMP/CMP kinase (UMP/CMPK)]. We also detected a 90% increase in glutathione S-transferase Class Pi, (GST-π), a protein critical for both free radical detoxification and also regulation of JNK-mediated signaling [80], [81].

Five proteins that function in cytoplasmic signaling and the regulation of these pathways were increase in exercised BDNF+/− mice compared to WT mice. In the striatum, we observed increased expression of the regulatory protein 14-3-3-protein epsilon in the STR of exercised BDNF +/− mice, as well as the enzymes MAPKK1, dual specificity phosphatase 3 (DUSP3), Hsp40 chaperone DnaJB6, and peptidylprolyl isomerase A (PPIA). In contrast, brain acyl-coenzyme A hydrolase, an enzyme involved in fatty acid metabolism, was decreased in the STR of exercised BDNF +/− mice, while phosphotyrosyl phosphatase activator (PTPA) was increased in the SN of WT mice. In addition to protein levels we observed a phosphorylation of calcineurin/protein phosphatase 3/protein phosphatase 2B in the SN of exercised WT mice.

Changes in expression levels of proteins involved in cytoskeleton and vesicle formation were observed in WT compared to BDNF+/− mice. There was an increase in the expression of neurocalcin delta (NCALD) and guanosine diphosphate dissociation inhibitor 2 (GDI2) in the STR of BDNF +/− mice and a decrease in the expression of internexin neuronal intermediate filament protein alpha (Ina) in the SN of BDNF +/− mice. In addition, a phosphorylation of N-ethylmaleimide sensitive fusion protein attachment factor alpha (NSF) was also noted.

## Discussion

BDNF has been shown to be critical for the survival of dopaminergic neurons throughout the brain, as well as for protection against toxin-induced cell death. In this study, our results support previous findings that BDNF haploisufficiency does not affect the normal development dopaminergic neurons in the SNpc [44]. Thus, the reduced expression of BDNF in these mice may have less of an impact on survival of DA neurons under normal conditions; but instead, as we show here, the deficiency in BDNF confers a significant vulnerability to toxic insult.

We show here that BDNF+/− mice ran the same amount as their WT littermates. This demonstrates that normal levels of BDNF are not required for normal activity levels. While some deficiencies in specific locomotor tasks have been reported in BDNF+/− mice [46], [47], our findings support previous literature showing that general activity in these mutant animals is not altered [42], [46]–[49], [78]. However, exercised BDNF happloinsufficient mice were not protected against MPTP-induced injury, as shown by the significant loss of SNpc DA neurons compared to WT mice allowed 90 days of voluntary exercise.

Numerous studies examining exercise-induced neuroprotection have suggested that neurotrophins, including BDNF, are critical for the induction of exercise-induced neuroprotection [15], [17], [82]. Support for the role of BDNF, specifically, includes studies demonstrating that neurons in the SNpc that express the BDNF receptor trkB are less sensitive to the toxic effects of MPTP than neurons that express the NT-3 receptor trkC [60]. In addition, implantation of fibroblasts engineered to overexpress BDNF significantly lessened MPP+ (the reactive metabolite of MPTP) toxicity in SNpc DA neurons [83]. In another study, concomitant administration of BDNF with MPP+ in non-human primates, not only decreased SNpc DA neuronal loss, but also enhanced dopaminergic reinnervation of the striatum and aided in the lessening of parkinsonian symptoms [84].

In this study we used the voluntary running paradigm rather than forced treadmill exercise because previous studies have suggested that enforced exercise may not be as potently neuroprotective as voluntary exercise since stress compromises many of the beneficial effects of exercise [85], including enhanced expression of BDNF [86]. Voluntary wheel running has been shown to induce greater increases in BDNF expression than forced or involuntary exercise [87]. Additionally, forced exercise has been shown to increase the release of corticosterone, a factor that suppresses the expression of BDNF mRNA and protein [88]–[90]. In contrast, activation of the stress response, as shown by an increase in corticosteroid, is not seen after voluntary wheel running [91], [92]. Voluntary exercise has also been shown to protect against the downregulation of BDNF induced by subsequent forced exercise [93]. Thus, the use of voluntary exercise in this study prevents possible dampening of the protective effects of exercise by stress.

The proteomic analysis revealed changes in several pathways that may underlie the lack of neuroprotection observed in exercised BDNF+/− mice. Protein changes were found in pathways associated with free radical detoxification, modulation of apoptosis through JNK signaling, amino acid transport/metabolism and energy metabolism. Each of these pathways have been implicated in the initiation and progression of PD [94], as well as exercise-mediated neuroprotection [16], [95]–[98].

Increased expression of proteins involved in amino acid transport and metabolism was observed in exercised BDNF+/− mice compared WT littermates, changes which suggest a higher level of oxidative stress in the transgenic mice. We observed an increase in the expression of flavin/biliverdin reductase B, an enzyme that converts biliverdin to the potent antioxidant bilirubin [99]. Increases were also seen in both the alpha type 4 and beta type 7 subunits of the proteasome, enzymes that function to degrade proteins using ATP/ubiquitin dependent processes. These latter changes suggest that there may be more damaged or mistranslated proteins in the BDNF+/− mice. One of the more interesting proteins that was upregulated in BDNF+/− mice is glutathione S-transferase, pi (GSTπ). This protein is a key antioxidant protein in the basal ganglia [100] and its expression has been shown to underlie sensitivity to MPTP [101]. In addition, relative levels of GSTπ are correlated with Parkinson’s disease progression [102]. Each of these proteins appears to respond to increases in oxidative stress [103], [104], and thus their increased abundance in exercised BDNF+/− mice compared to WT mice suggests a higher level of oxidative stress in exercised BDNF happloinsufficient mice. This potential increase in baseline oxidative stress would then contribute to an increased vulnerability to MPTP-induced oxidative stress that cannot be overcome by exercise in the BDNF +/− mice.

Several other proteins critical to signaling and gene regulation are also differently modulated in exercised BDNF+/− mice. The greatest changes were seen in phosphotyrosyl phosphatase activator (PTPA), 14-3-3-epsilon and DnaJB6. PTPA, which is decreased in the basal ganglia of exercised BDNF+/− mice compared to WT littermates, functions to regulate the expression of several proteins, including protein phosphatase 2A (PP2A) [105]. PP2A has been shown to be modulated by changes in tau and alpha-synuclein aggregation; both critical to the neuropathology of Parkinson’s disease [106]–[109].

DnaJB6 and 14-3-3 proteins were both upregulated in exercised BDNF+/− mice. 14-3-3 proteins have been functionally implicated in a number of cellular processes including transcription, biosynthesis, maintenance of the cytoskeleton, apoptosis and tumor suppression [110]. DnaJB6, a member of heat-shock protein 40 (HSP40) family [111], is highly expressed in Lewy bodies and astrocytes of parkinsonian patients but much more rarely expressed in cells from non-PD patients [112], [113]. Previous studies have shown that 14-3-3 is induced in the nigrostriatal pathway in response to the presence of misfolded proteins, including A53T alpha-synuclein [114]. It is also upregulated by the oxidative stress induced by complex I blockade that follows exposure to rotenone [115], a process that is similarly inhibited by MPTP [116], [117]. Oxidative stress has also been shown to upregulate expression of peptidylproline isomerase A [118], an effect that we also observed in exercised mice that were haploinsufficient for BDNF. Taken together, the upregulation of these factors in the exercised BDNF+/− mice suggests that BDNF+/− mice induce higher levels of oxidative stress than WT littermates after exposure to exercise.

Despite the fact that we observe higher expression of presumably protective proteins in BDNF+/− mice after 90 days of exercise, we do not see protection against MPTP-induced SNpc DA neuron death, suggesting that there are other factors that that have a significant impact on neuroprotection. For example, increased cellular energetics has been implicated in exercise-mediated neuroprotection [16], [95]. Exercised BDNF+/− mice showed lower expression of proteins involved in the glycolytic pathway compared to exercised WT mice. Dihydrolipoamide s-acetyltransferase was reduced by 60% in exercised BDNF+/−mice compared to WT mice. Reductions in this protein can lead to a buildup of lactate (primary lactic acidosis) in the brain and induce neuronal damage [119]. We also found a similar reduction in M2-type pyruvate kinase, which, in addition to its role in maintenance in cellular energy through ATP production [120], is also thought to act as a metabolic sensor, regulating cell growth, proliferation, and apoptosis [121]. Exercised BDNF+/− mice also showed reduced expression of the glycolytic enzyme alpha-enolase, which has been reported to also function as a neurotrophic factor [122], [123]. Additionally, the activity of this enzyme has been shown to modulate mitochondrial function and is reduced in the brains of mice carrying the A30P alpha-synuclein mutation [124]. One glycolytic enzyme, phosphoglycerate mutase 1 (PGM1) was upregulated in BDNF+/− basal ganglia. When glucose 6-phosphate is increased in the brain via depletion of glucose-6-phosphate dehydrogenase, NADH levels decrease, and subsequently there is an increase in MPTP-induced neuronal cell death [125]. Thus, the reduced expression of these proteins in the glycolytic pathway may compromise the resiliency of neurons against neurotoxic insult.

Exercised BDNF+/− mice also show significant decreases in proteins known to function in energy metabolism. We find that the exercised BDNF+/− mice have a 68% decrease in NADH dehydrogenase compared to WT mice. NADH dehydrogenase is the first enzyme of the mitochondrial electron transport chain, and disruption of this pathway has been implicated in Parkinson’s disease [126]. We also observed a decrease in expression of ATP synthase in exercised BDNF+/− mice. Increases in ATP synthase are thought to confer protection against oxidative stress [127]. Alterations in these two proteins suggest that deficits in complex I upregulation after exercise may underlie the lack of neuroprotection following induction of oxidative stress by MPTP administration in BDNF heterozygous mice.

In these studies we show that exercise does not protect dopaminergic neurons in the substantia nigra pars compacta from cell death following MPTP-induced oxidative stress in mice that are haploinsufficient for BDNF. Proteomic analysis indicates that in exercised BDNF+/− mice a number of proteins involved in cellular energetics pathways are not upregulated as compared to WT mice. Importantly, deficits in mitochondrial activity and energy pathways have been suggested to play a role in the initiation and progression of PD [94]. Reductions in the enzymatic activity of complex I enzymes [117] and mitochondrial abnormalities [128], [129] have been noted in human PD. Thus, the failure of the exercise to upregulate cellular energetic pathways in BDNF+/− mice may ultimately confer a greater susceptibility to neuronal death.

A number of clinical trials using BDNF for neuroprotection have had less than optimal results. One complicating factor is that because of the physical properties of BDNF, i.e. its size and charge, only small amounts will cross the blood-brain barrier, and thus requiring direct infusion into the brain [130]. However, in a few human subject studies, even short bouts of exercise were able to increase the expression of BDNF in serum [131]–[133] as well as induce genetic and epigenetic mechanisms that enhance BDNF expression [134]. In these studies we show that 90 days of voluntary exercise, an amount shown to be neuroprotective to oxidative stress induced by MPTP in C57BL/6 mice [16], [53] is not sufficient to protect dopaminergic neurons in the SNpc from MPTP in mice that are haploinsufficient for BDNF. This result highlights the critical importance of BDNF in exercise induced neuroprotection, independent of any other changes in other neurotrophic factors, and provides further support for the continued study of this neuroprotective protein.
